# Effect of a Community-Led Total Sanitation Intervention on the Incidence and Prevalence of Diarrhea in Children in Rural Ethiopia: A Cluster-Randomized Controlled Trial

**DOI:** 10.4269/ajtmh.20-0014cor

**Published:** 2024-07-03

In the original research paper, “Effect of a Community-led Total Sanitation Intervention on the Incidence and Prevalence of Diarrhea in Children in Rural Ethiopia: A Cluster-randomized Controlled Trial” by Cha and others (https://doi.org/10.4269/ajtmh.20-0014), the authors identified inconsistencies regarding the stated units in their raw data regarding prevalence rates.

The paper has therefore been corrected. The results and conclusions are unchanged. A summary of the corrected data is shown below:

1. Stated prevalence rate was changed from “The loss to follow-up rates were 95% for period prevalence and 93% for incidence and longitudinal prevalence” to “The follow-up rates were 95% for period prevalence and 93% for incidence and longitudinal prevalence. (in ABSTRACT)
2. Number of households with an improved toilet was changed from “The mean proportion of households with an improved toilet increased from 0.0% at baseline to 35.0% at 10 months after the CLTS initiation in the intervention group villages; however, increased from 0.5% to 2.8% in the control group villages (risk difference, 32.3%; 95% CI, 19.1–45.4%; *P* < 0.001)…” to “The mean proportion of households with an improved toilet increased from 0.0% at baseline to 35.0% at 10 months after the CLTS initiation in the intervention group villages; however, it increased from 0.7% to 2.8% in the control group villages (risk difference, 32.3%; 95% CI, 19.1–45.4%; *P* < 0.001)…” (page 536)
3. Improved toilet coverage data was changed from “At the 10-month follow-up, 4 of the 24 intervention villages had improved toilet coverage of 70% or greater. In the control villages, no community had coverage of 30% or greater. Ownership of a partially improved household toilet (defined as having a pit, pit hole cover, and slab)…” to “At the 10-month follow-up, 4 of the 24 intervention villages had improved toilet coverage of 70% or greater. In the control villages, no community had coverage of 30% or greater. Ownership of a partially improved household toilet (defined as having a pit, pit hole cover, and slab)…” (page 536)
4. Significance value for “Feces around pit hole” was changed from “0.20” to “0.02” *P*-value. (TABLE 5, p. 541)

